# Oral-gut axis in inflammation: periodontitis exacerbates ulcerative colitis via microbial dysbiosis and barrier disruption

**DOI:** 10.1186/s12903-025-06269-8

**Published:** 2025-06-03

**Authors:** Jinping Yu, Jinglu Lyu, Tongxin Zhu, Yang Li, Hanping Xia, Qing Liu, Lili Li, Bin Chen

**Affiliations:** Department of Periodontology, Nanjing Stomatological Hospital, Affiliated Hospital of Medical School, Institute of Stomatology, Nanjing University, Nanjing, China

**Keywords:** Periodontitis, Ulcerative colitis, Dextran sodium sulfate, Immune response, Gut microbiota, Intestinal barrier

## Abstract

**Background:**

Periodontitis is a chronic inflammatory disease, having significant impact on systemic conditions. Ulcerative colitis (UC) is a chronic relapsing inflammatory disorder of the intestines. Studies have suggested a potential association between periodontitis and UC. This study aims to elucidate the influence of periodontitis on the progression of UC and to uncover the potential mechanistic pathways involved.

**Methods:**

A total of 20 male C57BL/6J mice were randomly assigned to four groups: Sham, Periodontitis (P), UC, and Periodontitis + UC (P-UC). A chronic UC model was induced by alternating oral administration of 1% and 0.5% Dextran Sulfate Sodium Salt (DSS) solution, while periodontitis was induced by ligatures. Disease severity was accessed using Disease Activity Index (DAI), histopathology, and intestinal permeability assays. Gut microbiota and periodontal microbiota was analyzed using 16S rRNA sequencing. Tumor necrosis factor-alpha (TNF-α), interleukin-6 (IL-6) were measured by quantitative PCR (qPCR) to evaluate the systemic inflammation burden. Zonula occludens-1 (ZO-1) and occludin in intestinal tissues were assessed using qPCR and immunohistochemistry. Correlation analyses were performed between periodontal destruction indices and markers.

**Results:**

A chronic UC model closely resembling clinical conditions was successfully established. The P-UC group exhibited earlier and more pronounced body weight loss than the UC group. Colonic inflammation was exacerbated, with significantly elevated TNF-α and IL-6 expression (*P* < 0.05). In the P-UC group, intestinal barrier disruption was evident with reduced occludin protein levels (*P* < 0.01) and increased intestinal permeability (*P* < 0.05), indicated by serum diamine oxidase (DAO). Both the P-UC and UC groups exhibited notable dysbiosis of the gut microbiota, with the P-UC group showing significantly higher abundance of UC-associated bacteria, such as *Muribaculum* and *Allobaculum* (*P* < 0.05), compared to the UC group. A trend toward reduced abundance of the gut-protective bacterium *Akkermansia* was also observed (*P* = 0.06). Pearson correlation analysis confirmed the association between periodontitis and intestinal inflammation, suggesting that intestinal barrier dysfunction and gut microbiota dysbiosis may be key mediators in periodontitis-induced UC exacerbation.

**Conclusion:**

Periodontitis may exacerbate UC by increasing harmful gut bacteria, reducing beneficial bacteria, and promoting the secretion of pro-inflammatory cytokines, thereby disrupting the intestinal barrier and worsening UC severity.

**Graphical abstract:**

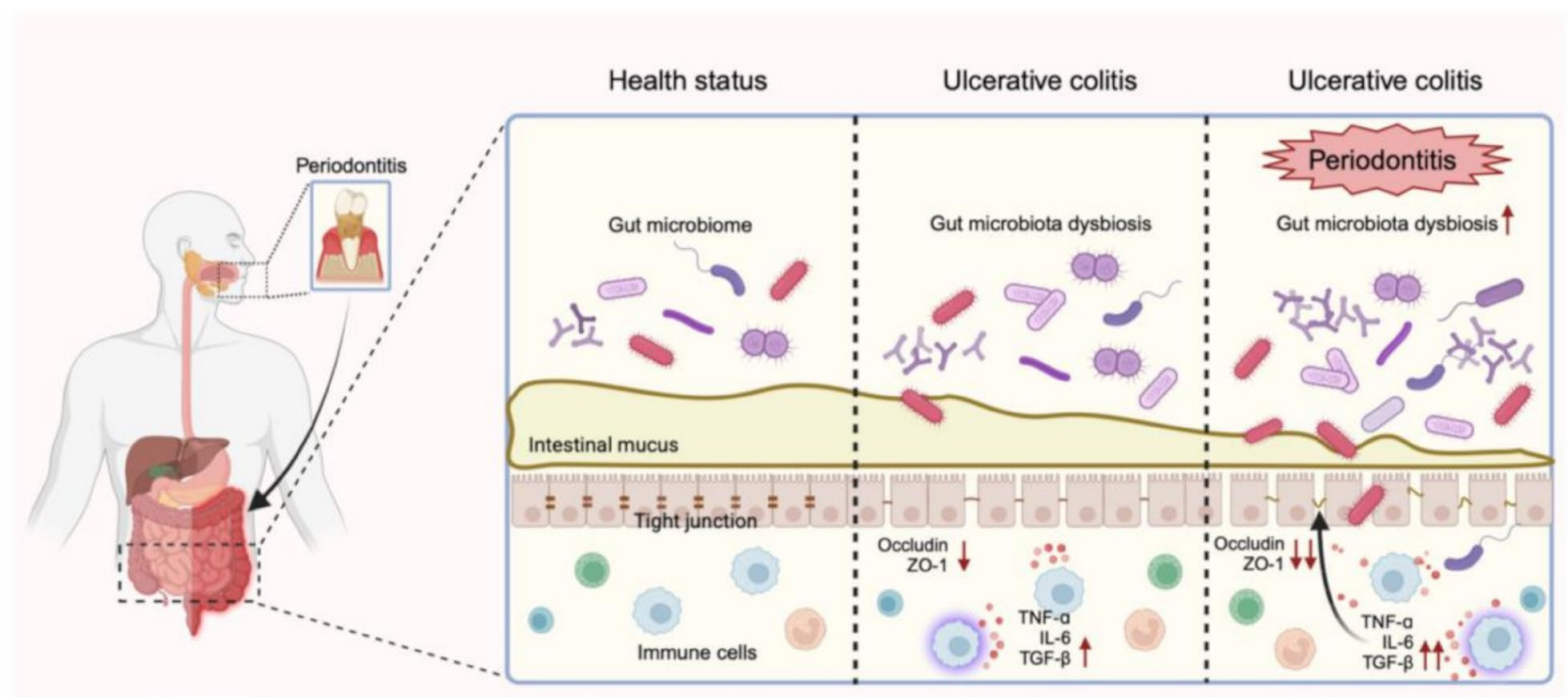

**Supplementary Information:**

The online version contains supplementary material available at 10.1186/s12903-025-06269-8.

## Introduction

Periodontitis is a chronic inflammatory disease triggered by plaque microorganisms. Epidemiological studies indicate that periodontitis ranks as the sixth most prevalent disease globally, affecting over 10% of the population worldwide [1]. The disease can progress over decades and is the leading cause of tooth loss in adults [2]. Over the past two decades, growing research has highlighted on the significant impact of periodontitis on systemic conditions, including diabetes, cardiovascular disease and inflammatory bowel disease (IBD).

IBD is a chronic, relapsing inflammatory disorder of the intestines, encompassing Crohn’s disease (CD) and ulcerative colitis (UC). It follows a cyclical pattern of active and remission phases, with active phases characterized by abdominal pain, diarrhea, and bloody stools. Moreover, research suggests that IBD may progress to colorectal cancer [3]. Effective treatments for IBD remain limited, and frequent relapses severely impact patients’ quality of life. The prevalence of IBD is rising rapidly, affecting an estimated 3.5–4.2 million people in developed countries such as North America and Europe, where it now exceeds 0.5%. While newly industrialized countries currently have lower prevalence rates, incidence is increasing sharply [4]. Given this trend, it is crucial to control and eliminate IBD risk factors.

Numerous clinical studies have shown that periodontitis may be a significant risk factor for IBD [5], as it can exacerbate existing intestinal inflammation and complicate disease management. A recent meta-analysis reported associations between periodontitis and both UC and CD, with odds ratios of 5.37 and 3.64, respectively [6]. Beyond cross-sectional studies, retrospective and cohort studies also suggest this link. One cohort study found that periodontitis patients exhibited a 1.56-fold higher risk of developing UC [7]. Similarly, a large-scale retrospective study involving one million participants identified a significantly higher UC risk among those with periodontitis [8]. However, some research indicates no significant association between the two conditions [9], highlighting the need for further research to clarify their relationship and underlying mechanisms.

To further explore the impact of periodontitis on UC, this study utilized a UC mouse model induced by alternating 1% and 0.5% dextran sulfate sodium (DSS) solution. This model approximates the actual chronic clinical course experienced by UC patients more closely, aiming to elucidate the connection between periodontitis and UC, explore potential mechanisms, and contribute to future research on UC pathogenesis and clinical management.

## Materials and methods

### Experimental design

Seven-week-old male C57BL/6J mice were purchased from Beijing Vital River Laboratory Animal Technology Co., Ltd. and housed under specific pathogen-free (SPF) conditions at the Laboratory Animal Center of Nanjing Agricultural University. All animal experiments adhered to ethical guidelines and were approved by the Laboratory Animal Welfare and Ethics Committee of Nanjing Agricultural University (No. PZW2024032, China). Based on our previous work and existing literature in the field [10, 11], and in adherence to the 3Rs principle of animal welfare (Replacement, Reduction, and Refinement), we chose to use 5 mice per group to ensure both ethical responsibility and adequate statistical power.

After one week of acclimatization, 20 mice were randomly assigned to four groups: (1) group Sham(*n* = 5), receiving regular drinking water for 30 days; (2) group P(*n* = 5), subjected to ligature-induced periodontitis starting on day 7 and maintained for the following 23 days; (3) group UC(*n* = 5), in which UC was induced by receiving alternating 1% and 0.5% w/v DSS for 30 days; and (4) group P-UC(*n* = 5), receiving the same DSS regimen as the UC group, with the same ligature-induced periodontitis as the P group.

After grouping, animals in each group were not allowed to enter cages belonging to the other groups. All lids, cages, bedding, bottles, and water were sterilized before use. Animals severely injured from fighting or having lost teeth with periodontitis were excluded.

### Induction of periodontitis and chronic colitis

Experimental periodontitis was induced by ligature. Briefly, mice were anesthetized via intraperitoneal injection of tribromoethanol (Jitian, China) according to body weight. After achieving full anesthesia, 5 − 0 silk sutures (Jinhuan, China) was ligated around the left maxillary second molars [12]. Following the procedure, mice were placed on a heating pad for recovery. The ligatures were checked every other day. Once they fell off, the tooth was ligated again immediately.

The chronic ulcerative colitis model was induced by administering 1% w/v dextran sulfate sodium (DSS; MP Biomedical) in drinking water. Mice received 1% DSS for 16 days, followed by 0.5% DSS for 7 days, and then 1% DSS for another 7 days [13].

### Sample collection

Mice were anesthetized via intraperitoneal injection of 1.25% tribromoethanol (0.2 mL/10 g body weight). Blood samples (∼ 0.5 mL) were collected from the orbital venous sinus. After blood sampling, mice were anesthetized via intraperitoneal injection of sodium pentobarbital (150 mg/kg). After confirming full unconsciousness, mice were humanely euthanized by cervical dislocation under anesthesia. Following euthanasia, the maxillary bone and surrounding tissues were harvested and fixed in 4% neutral paraformaldehyde. Ligatures were collected from the P and P-UC groups, while in the Sham and UC groups, sterile 5 − 0 silk sutures were briefly inserted into the gingival sulcus before removal. All samples were stored at -80 °C. The thoracic and abdominal cavities were opened to collect the cecum and proximal colon, measuring their distance and weight. Approximately 2 mm of proximal colon tissue was preserved in RNA stabilization solution, while a 5 mm colon segment was washed and fixed in 4% neutral paraformaldehyde. Cecal contents were stored at -80 °C. To minimize bias, group allocation and treatment were performed by a single unblinded investigator. All subsequent sample collection, tissue processing, and outcome assessments were conducted by personnel blinded to group identity. Histological evaluation was performed by two independent, blinded pathologists.

### Micro–Computed tomography analysis

Maxillae were fixed in 4% paraformaldehyde for 72 h before micro–Computed Tomography (CT) scanning (SkyScan 1172, Bruker, Belgium). The scanning voxel resolution was 18 μm. Two-dimensional images were reconstructed into three-dimensional models using NReconstruction (version 1.4.4, Bruker). The distance from the cemento-enamel junction to the alveolar crest was measured using DataViewer (version 1.5.4.0, Bruker) to assess bone resorption.

### Histological analysis of periodontal tissue

The maxillae were decalcified in 10% EDTA solution (pH 7.2; Servicebio), with the solution changed every 3 days for 4 weeks. Sections were then prepared for hematoxylin and eosin (HE) staining. Images were captured using a slide scanner and analyzed with CaseViewer software.

### Intestinal microbiota DNA extraction and 16S rRNA gene sequencing

DNA was extracted from cecal contents using a fecal DNA extraction kit (OMEGA Soil DNA Kit (M5635-02), Omega Bio-Tek, Norcross, GA, USA). The integrity and quantity of extracted DNA were initially verified by 2% agarose gel electrophoresis and purified using an AxyPrep DNA gel extraction kit. PCR amplification of the 16S rRNA gene (V3-V4 region) was performed using barcoded universal primers 338 F (5′-ACTCCTACGGGAGGCAGCA-3′) and 806R (5′-GGACTACHVGGGTWTCTAAT-3′). Amplification products were confirmed via 2% agarose gel electrophoresis and subsequently purified using a silica column-based gel extraction kit. Sequencing libraries were constructed without additional PCR enrichment to minimize amplification bias, and library quality was assessed using a Bioanalyzer system. Paired-end sequencing (2 × 250 bp) was conducted on an Illumina MiSeq platform, yielding high-throughput data for downstream analysis of gut microbial community structure.

### Quantification of Short-chain fatty acids (SCFAs) by gas Chromatography-Mass spectrometry (GC-MS)

Samples were homogenated for 1 min with 500 µL of water and 100 mg of glass beads, and then centrifuged at 4℃ for 10 min at 12,000 rpm. 200 µL supernatant was extracted with 100 µL of 15% phosphoric acid and 20 µL of 375 µg/mL 4-methylvaleric acid solution as IS and 280 µL ether. Subsequently, the samples were centrifuged at 4℃ for 10 min at 12,000 rpm after vortexing for 1 min and the supernatant was transferred into the vial prior to GC-MS analysis. The GC analysis was performed on trace 1310 gas chromatograph (Thermo Fisher Scientific, USA). The GC was fitted with a capillary column Agilent HP-INNOWAX (30 m × 0.25 mm ID × 0.25 μm) and helium was used as the carrier gas at 1 mL/min. Injection was made in split mode at 10:1 with an injection volume of 1 µL and an injector temperature of 250℃. Mass spectrometric detection of metabolites was performed on ISQ LT (Thermo Fisher Scientific, USA) with electron impact ionization mode. Single ion monitoring (SIM) mode was used with the electron energy of 70 eV. Extracted-ion chromatograms for each ion were abstracted based on the information of diagnostic ions and quantification ions. Peak areas would be detected by retention times for each compound. The calibration curves were obtained as plots of the peak area ratio of the target compounds to an internal standard versus the target compound concentration and calculate the concentration in samples.

### Bioinformatic analysis

Raw sequencing data were processed using QIIME (version 1.9.1, University of Colorado, USA). Paired-end reads were assembled, quality-controlled, and filtered. Sequences were assigned to operational taxonomic units (OTUs) at 97% similarity using UPARSE (version 7.1). Chimeric sequences were removed using UCHIME. Taxonomic classification was performed using RDP Classifier with the Greengenes2 database (70% confidence threshold). Bray-Curtis distances were calculated for community structure analysis. Statistical comparisons were conducted using the Wilcoxon rank-sum test. Linear discriminant analysis (LDA) was performed to identify differential taxa, with results visualized using R (version 3.2.5, University of Auckland, New Zealand).

### Statistical analysis

Data analysis was performed using SPSS 28 (IBM, NY, USA) and GraphPad Prism 9 (GraphPad Software, CA, USA). Results are presented as mean ± standard deviation. Group comparisons were conducted using independent t-tests and Wilcoxon rank-sum tests. For multi-group comparisons, one-way ANOVA followed by post-hoc multiple comparisons or Kruskal-Wallis tests were applied. Pearson correlation analysis was used to assess associations between variables. A *p*-value < 0.05 was considered statistically significant. All graphical representations were generated using GraphPad Prism 9.

## Results

### Establishment of the periodontitis model and periodontal tissue conditions in each group

**Fig. 1:**
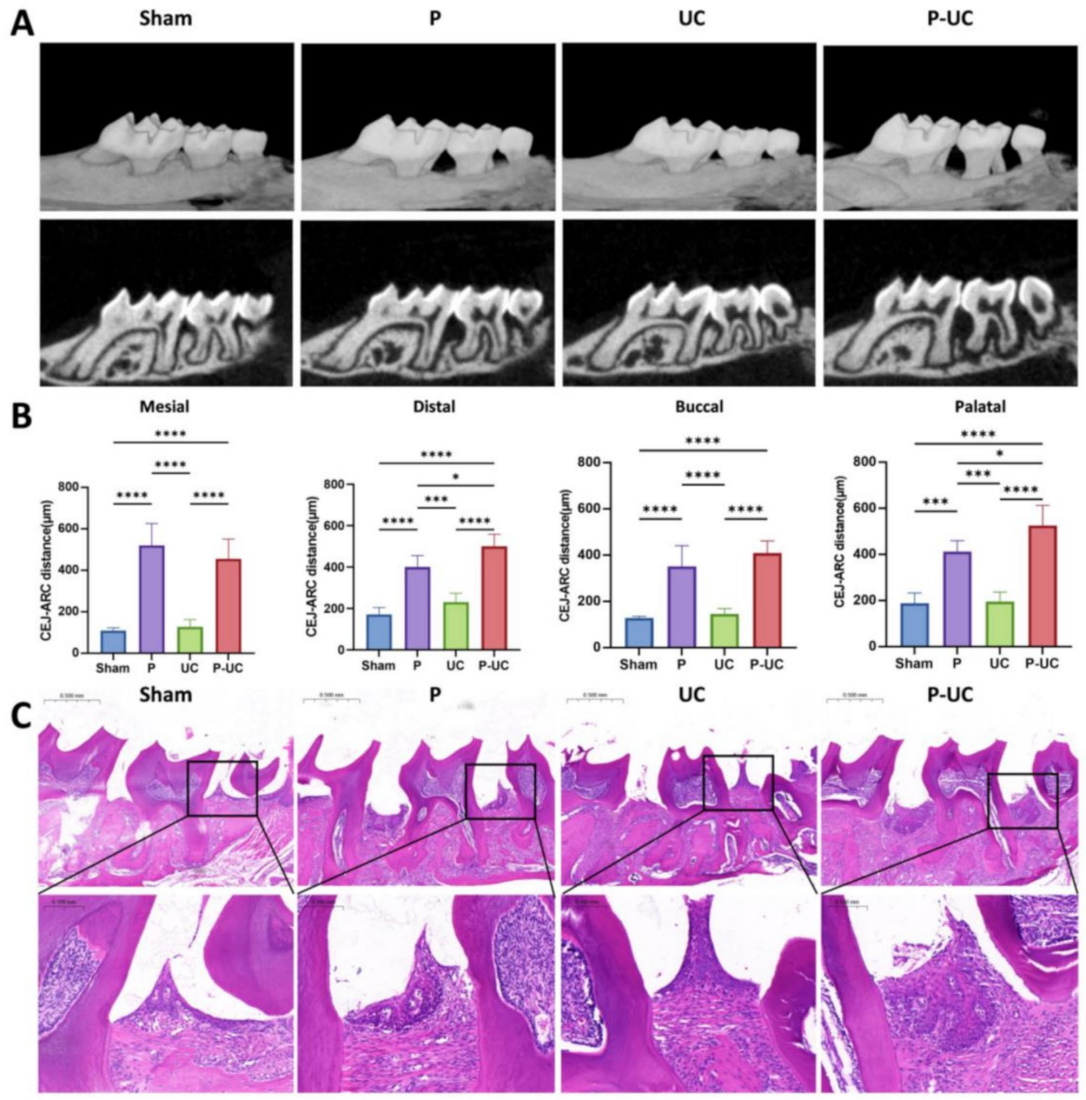
Establishment of the periodontitis model and periodontal tissue inflammation in each group. (**A**) Micro-CT scans of the maxilla in each group, showing alveolar bone resorption around the second maxillary molars in the P and P-UC groups; (**B**) Measurement of the distance from the cementoenamel junction (CEJ) to the alveolar crest (ARC) at mesial, distal, buccal, and palatal sites of the second maxillary molars in each group. The distances in the P and P-UC groups were significantly greater than in the Sham group (*P* < 0.0001), and the distal and palatal distances in the P-UC group were significantly greater than in the P group (*P* < 0.05); (**C**) Representative H&E-stained histological sections of the second maxillary molars in each group. *n* = 5, * *P* < 0.05, *** *P* < 0.001, **** *P* < 0.0001. Sham, sham-operated group; P, periodontitis group; UC, UC group; P-UC, periodontitis + UC group. CEJ to ARC: distance from CEJ to ARC

The ligatures remained intact throughout the experiment in both the P and P-UC groups. Micro-CT analysis revealed significant alveolar bone resorption around the maxillary second molars in both P and P-UC groups, with greater distances from the alveolar crest to the cementoenamel junction (CEJ) at the mesial, distal, buccal, and palatal sites compared to the Sham group (*P* < 0.0001), confirming the successful establishment of the periodontitis model (Fig. 1A, B). H&E staining further validated the model, showing gingival recession below the CEJ and inflammatory cell infiltration in the gingiva of both the P and P-UC groups (Fig. 1C).

### Effects of periodontitis on chronic UC

#### Effect on UC severity

**Fig. 2:**
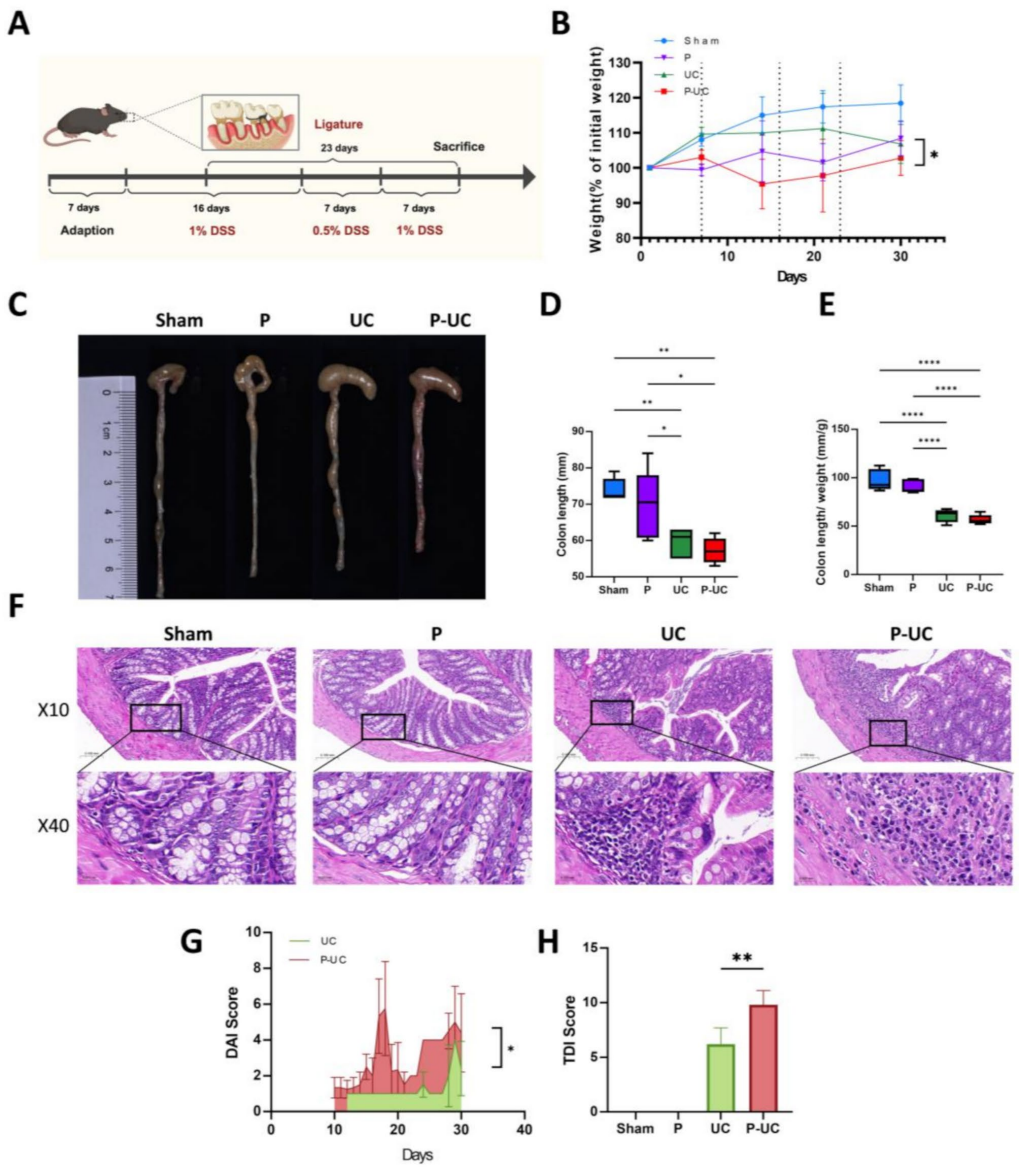
Effect of periodontitis on the severity of UC. (**A**) Experimental workflow of periodontitis affecting UC; (**B**) Percentage change in body weight of mice in each group. By the end of the experiment, the body weight of the P-UC group had significantly decreased compared to the UC group (*P* < 0.05); (**C**) Representative images of colonic morphology and length in each group, showing edema, congestion, and changes in intestinal contents in the UC and P-UC groups; (**D**) Colon length in each group, showing a significant reduction in the UC and P-UC groups; (**E**) Colon length-to-weight ratio in each group; (**F**) Representative H&E-stained sections of the colon in each group; (**G**) DAI scores assessing the general condition of mice, indicating more severe disease in the P-UC group compared to the UC group; (**H**) TDI scores for colonic histological damage assessment, showing more severe damage in the P-UC group compared to the UC group. *n* = 5, * *P* < 0.05, ** *P* < 0.01, ***P < 0.001, ****P < 0.0001. TDI, Tissue Damage Index

The experimental workflow of periodontitis affecting UC is shown in Fig. 2A. Body weight is a key indicator of UC severity. The percentage change in body weight throughout the experiment is presented in Fig. 2B. The P-UC group exhibited an earlier onset of weight loss compared to the UC group. Due to the use of a low-concentration DSS solution to induce chronic UC, weight loss occurred gradually. By the end, body weight in the P-UC group was significantly lower than in the UC group (*P* < 0.05). Additionally, disease severity, as assessed by the DAI index, was significantly higher in the P-UC group compared to the UC group (*P* < 0.05) (Fig. 2G). H&E staining revealed pronounced colonic inflammation in both the UC and P-UC groups, with significantly higher tissue damage index (TDI) scores in the P-UC group (*P* < 0.01) (Fig. 2F and H). Both groups exhibited colon edema, congestion, altered intestinal content consistency, and significantly shortened colon length, indicative of colonic inflammation (Fig. 2C and D). The colon length-to-weight ratio, a measure of edema severity, was significantly lower in both the UC and P-UC groups compared to the Sham and P groups, though no significant difference was observed between the UC and P-UC groups (Fig. 2E).

#### Effects on colonic inflammatory factors and intestinal barrier function

**Fig. 3:**
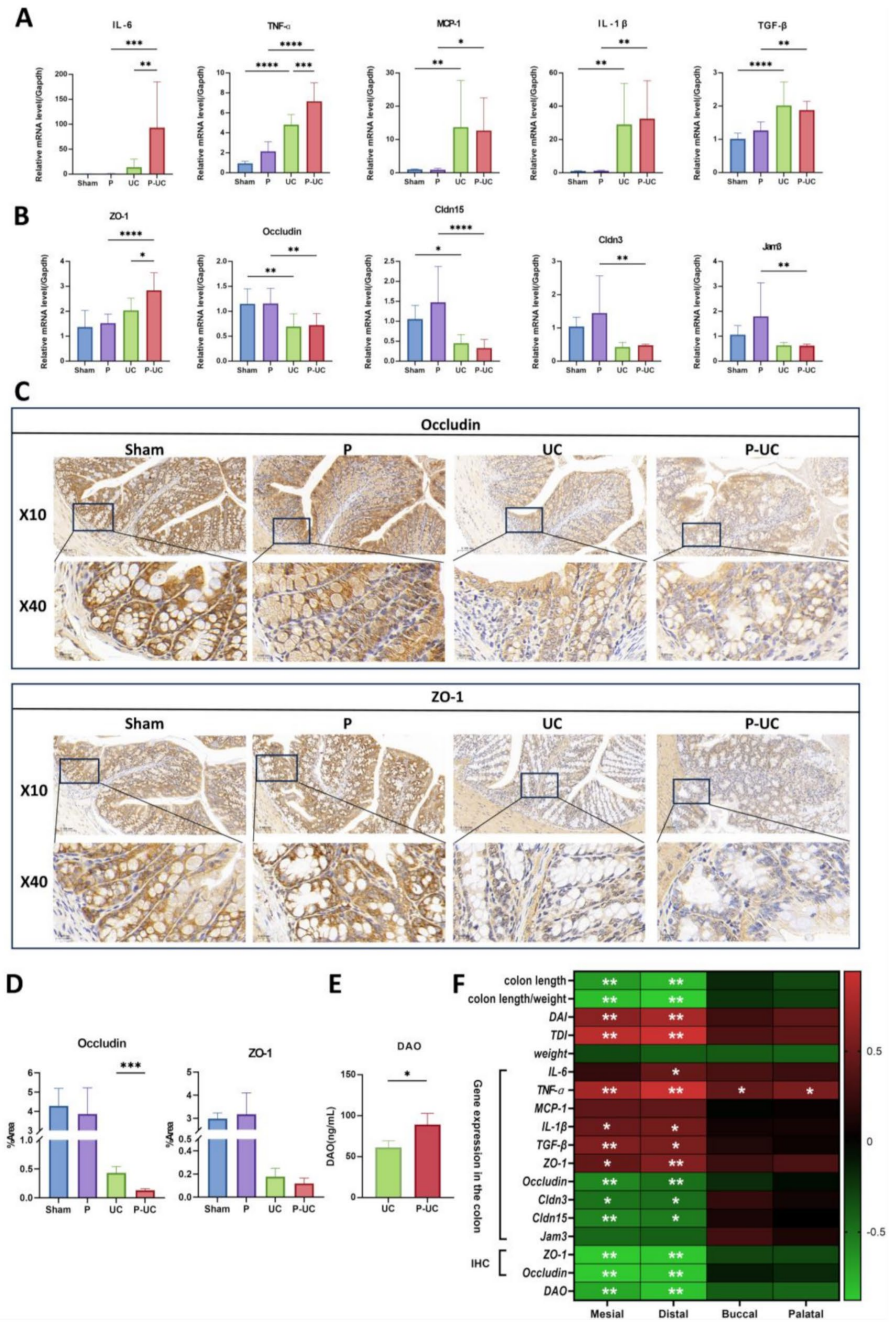
Effects of periodontitis on colonic inflammation and intestinal barrier integrity. (**A**) Gene transcription levels of inflammatory cytokines in colonic tissue. The mRNA levels of TNF-α and IL-6 were significantly elevated in the P-UC group compared to the UC group (*P* < 0.001, *P* < 0.01). MCP-1, IL-1β, and TGF-β mRNA levels were significantly increased in the UC and P-UC groups compared to the Sham and P groups; (**B**) Gene transcription levels of tight junction proteins in colonic tissue. ZO-1 expression was significantly upregulated in the P-UC group compared to the UC group (*P* < 0.05), while Occludin, Cldn3, Cldn15, and Jam3 mRNA levels were decreased in the UC and P-UC groups compared to the Sham and P groups; (**C**) Representative immunohistochemical staining images of ZO-1 and Occludin in colonic tissue; (**D**) Semi-quantitative analysis of ZO-1 and Occludin staining, showing a significant decrease in Occludin expression in the P-UC group (*P <* 0.001); (**E**) DAO levels were significantly elevated in the P-UC group compared to the UC group(*P <* 0.05); (**F**) Pearson correlation analysis between periodontitis and intestinal inflammation (*P <* 0.01). *n* = 5, *P < 0.05, **P < 0.01, ***P < 0.001, ****P < 0.0001. IL-6, Interleukin-6; TNF-α, Tumor Necrosis Factor Alpha; MCP-1, Monocyte Chemoattractant Protein-1; ZO-1, Zonula Occludens-1; Cldn15, Claudin 15; Jam3, Junctional Adhesion Molecule-3; DAO, diamine oxidase

qPCR analysis revealed significantly elevated mRNA levels of inflammatory markers MCP-1, IL-1β, and TGF-β in colonic tissues of both the UC and P-UC groups compared to the Sham and P groups. Additionally, the P-UC group exhibited significantly higher TNF-α and IL-6 transcription levels compared to the UC group (*P* < 0.01) (Fig. 3A). Tight junction proteins expression in colonic tissues is shown in Fig. 3B. Compared to the Sham and P groups, mRNA levels of Occludin, Cldn3, Cldn15, and Jam3 were reduced in both UC and P-UC groups. However, ZO-1 mRNA levels were significantly higher in the P-UC group compared to the UC group (*P* < 0.05). Immunohistochemical staining indicated more severe intestinal barrier disruption in the P-UC group, with significantly reduced Occludin protein levels (*P* < 0.05) (Fig. 3C and D). ELISA results further confirmed this disruption, showing significantly elevated serum diamine oxidase (DAO) levels in the P-UC group (*P* < 0.05) (Fig. 3E). Pearson correlation analysis revealed a significant correlation between periodontitis and intestinal inflammation (*P* < 0.01) (Fig. 3F). Further analysis indicated that disruptions in the intestinal barrier and gut microbiota may be critical mediators of periodontitis-induced UC exacerbation (Figure S2A, B).

### Periodontitis-induced gut microbiota dysbiosis and intestinal metabolic changes in UC mice

#### Periodontal microbiota composition

Compared to the Sham group, the P, UC, and P-UC groups exhibited significantly decreased α-diversity indices, including Chao1, Observed-species, and Faith-pd. Notably, the P-UC group had a significantly lower Faith-pd index compared to the UC group (Figure S1A). Principal coordinate analysis (PCoA) based on Bray-Curtis distances revealed distinct shifts along the PC1 axis in the UC and P-UC groups compared to the Sham group, with the P and P-UC groups clustering more closely (Figure S1F). Clear separation was also observed between the UC and P-UC groups (Figure S1C). Differential species and composition analysis of periodontal microbiota in each group were displayed in Figure S1B, S1D and S1E. At the genus level, the P-UC group exhibited an increased abundance of *Ligilactobacillus* (*P* < 0.05) and a decreased abundance of *Allobaculum* (*P* < 0.01) compared to the UC group (Figure S1G).

#### Gut microbiota analysis

**Fig. 4:**
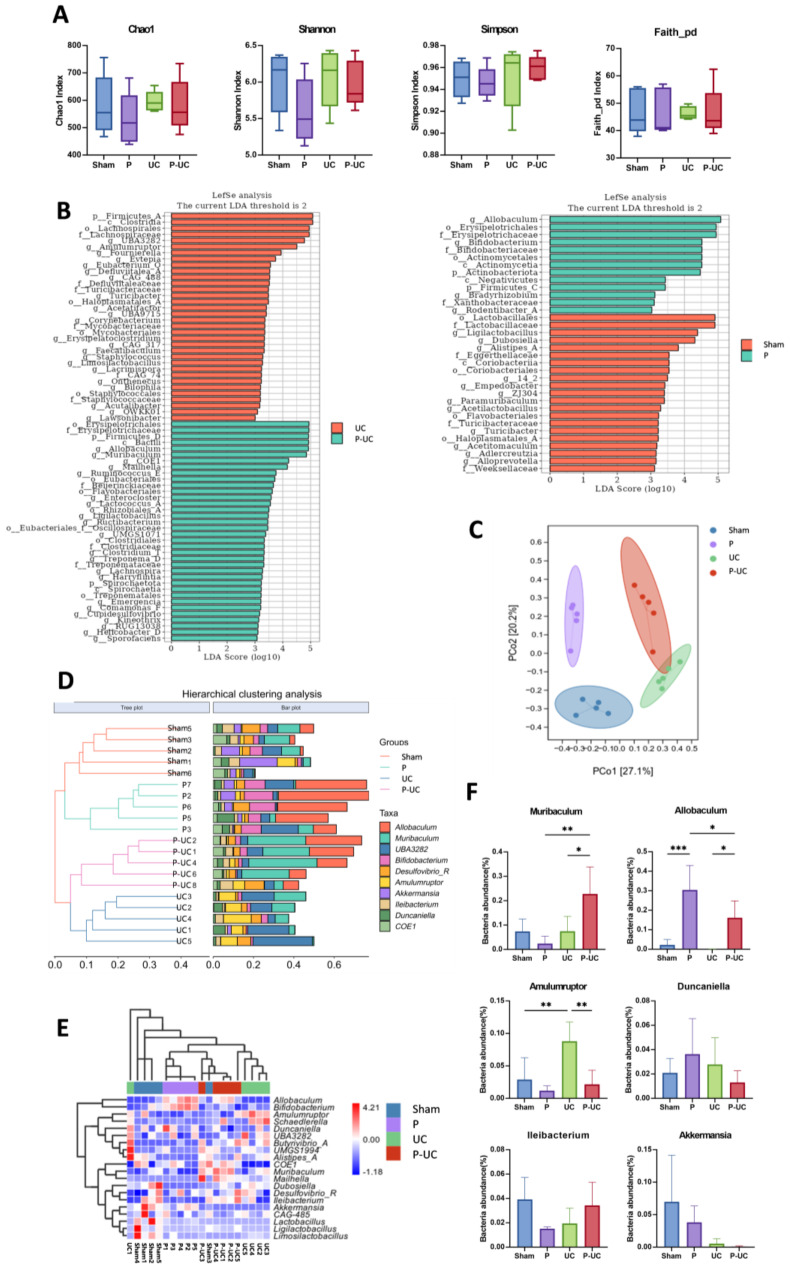
Effects of periodontitis on gut microbiota in mice. (**A**) ɑ-diversity indices of gut microbiota in each group, showing no significant differences; (**B**) Differential species and biomarker analysis of gut microbiota in each group; (**C**) β-diversity distance analysis of gut microbiota, showing clear separation among the four groups; (**D**) Hierarchical clustering analysis of the top 10 most abundant genera in gut microbiota, showing distinct clustering among groups; (**E**) UPGMA clustering heatmap of the top 20 most abundant genera in gut microbiota; (**F**) Quantitative analysis of gut microbiota at the genus level. The abundance of *Muribaculum* and *Allobaculum* significantly increased, while *Amulumruptor* significantly decreased in the P-UC group compared to the UC group. The abundance of *Akkermansia* was reduced in the UC and P-UC groups compared to the Sham group. *n* = 5, *P < 0.05, **P < 0.01, ***P < 0.001, ****P < 0.0001

The α-diversity analysis showed no significant differences among groups (Fig. 4A). However, PCoA based on Bray-Curtis distances revealed a clear stratification of gut microbial communities across the four experimental group, reflecting substantial compositional differences in their microbiota. Differential species and composition analysis of gut microbiota in each group were displayed in Fig. 4B and E. Notably, the P-UC, the UC and P groups diverge significantly from the Sham group, emphasizing the specific alterations in gut microbial composition induced by each experimental condition (Fig. 4C). A clustering tree analysis confirmed well-separated groupings, indicating significant differences in β-diversity (Fig. 4D). At the genus level, compared to the UC group, the P-UC group exhibited a significantly increased abundance of *Muribaculum* and *Allobaculum* (*P* < 0.05) and a significantly decreased abundance of *Amulumruptor* (*P* < 0.01). The P-UC group showed a reduced abundance of *Akkermansia* compared to the Sham group (*P* = 0.06), with no significant differences observed for other genera (Fig. 4F).

#### Detection of SCFAs in intestinal content

Short-chain fatty acids (SCFAs) profiling of intestinal contents revealed that acetate, butyrate, and propionate were the predominant SCFAs, accounting for approximately 60%, 20%, and 18% of total SCFAs content, respectively (Figure S3A). Compared to the Sham and P groups, the UC and P-UC groups exhibited increased levels of hexanoic acid, although the differences were not statistically significant (Figure S3B). SCFAs composition in each group is presented in Figure S3C. Pearson correlation analysis revealed a significant correlation between SCFAs and TGF-β (*P* < 0.05) (Figure S3D).

## Discussion

In this study, we successfully established a new chronic UC model and compared it with acute and conventional chronic UC models. This novel model more accurately mimicked clinical UC features, especially the alternating active and remission phases, and revealed that periodontitis may exacerbate UC, likely through elevated inflammatory factors (TNF-α, IL-6) and gut microbiota alterations.

### Establishing UC models more relevant to clinical reality

Current acute UC models which are widely used in mice employ 3% DSS for 7–10 days. While they replicate some acute pathological features but fail to reflect the alternating active and remission phases characteristic of the disease [14, 15] making them unsuitable for investigating the long-term impact of periodontitis on intestinal inflammation.

Chronic colitis models involve either prolonged administration of low-dose DSS or repeated cyclic exposure to DSS, which typically consists of 7 days of 2–5% DSS treatment followed by 14 days of water, repeated for 4–8 cycles [14]. Unlike acute models, chronic inflammation in these models is characterized by mononuclear leukocyte infiltration, crypt architectural disarray, increased lymphocytic infiltration in the submucosa, and an increased crypt base-to-muscularis mucosae distance [16, 17]. However, during alternating administration of DSS solution and water, higher DSS concentrations may results in increased mortality, and mice tend to recover rapidly during water consumption periods, limiting long-term intestinal inflammation.

To address these limitations, we developed a novel chronic UC model using alternating 1% and 0.5% DSS solutions. This approach more accurately simulated clinical UC cycles while improving the animal survival. HE staining of colonic tissues in both UC and P-UC groups confirmed that the newly established model closely resembles the histopathological features of chronic UC models [18–20]. Importantly, the 0.5% DSS solution maintained a chronic inflammatory state during remission phases, enabling the investigation of periodontitis-driven effects under sustained UC burden. Additionally, the lower DSS concentrations reduced colonic damage, potentially amplifying the effects of periodontitis on UC while also serving as a valuable reference for future model selection in studies examining the systemic impact of periodontitis.

### Periodontitis exacerbates UC

Body weight loss, a key UC model indicator, was greater in the P-UC group than in the UC group, along with higher DAI scores, suggesting more severe colonic inflammation. Furthermore, the colonic inflammation is associated with immune cell infiltration, enhanced inflammatory response, and gut microbiota dysbiosis [21]. Histological analysis and elevated levels of pro-inflammatory cytokines, along with increased intestinal permeability, further support the notion that periodontitis aggravates UC. Correlation analysis also revealed a significant positive association between the two conditions. Further analysis indicated that disruptions in the intestinal barrier and gut microbiota may be critical mediators of periodontitis-induced UC exacerbation, as demonstrated by the following findings:

### Possible pathways by which periodontitis exacerbates UC

#### Intestinal barrier disruption via inflammatory factors

TNF-ɑ and IL-6 are pivotal mediators of intestinal inflammation and intestinal barrier dysfunction, significantly driving the onset and progression of UC [22]. Previous studies have reported elevated levels of proinflammatory cytokines in the intestinal tissues and serum of UC patients [18, 20, 23–25]. Furthermore, monoclonal antibody therapies targeting these cytokines have demonstrated significant clinical efficacy in UC treatment [26]. In this study, we observed marked upregulation of TNF-ɑ and IL-6 mRNA expression in the P-UC group, indicating an amplified inflammatory response in the presence of periodontitis.

The intestinal barrier, crucial for defending against pathogens, is often compromised in UC, leading to immune dysregulation. Previous studies suggest that periodontitis may contribute to intestinal barrier integrity [27]. TNF-ɑ and IL-6 can disrupt tight junction proteins, compromise mucosal integrity, and trigger immune activation [28]. Additionally, monoclonal antibody therapy has been demonstrated to mitigate UC-induced colonic damage and restore intestinal permeability [29]. Consistent with prior studies, our study indicated more severe intestinal barrier disruption in the P-UC group. These findings suggest that periodontitis may exacerbate UC through intensified cytokine signaling, leading to further barrier breakdown and inflammation.

#### The role of gut microbiota

As the largest microbial community in the human body, gut microbiota plays a crucial role in maintaining physiological homeostasis, regulating immune responses, aiding nutrient digestion, and protecting against pathogenic invasion [30, 31]. Disruptions in gut microbiota composition can disturb host-microbe equilibrium, contributing to the development of UC and irritable bowel syndrome. Our previous research showed that saliva from periodontitis patients can reshape the gut microbiome [32]. 16S rRNA sequencing revealed abnormal bacterial aggregation and compositional shifts in both UC and periodontitis, with their coexistence intensifying dysbiosis.

Gut dysbiosis is a key contributor to UC, with certain bacterial genera influencing inflammation via microbial abundance and metabolite production. Our analysis identified a significant enrichment of *Allobaculum* and *Muribaculum* in the P-UC group, which genera were reported being associated with metabolic syndrome and intestinal inflammatory diseases in both humans and mice [33].

The intestinal mucosal epithelium is shielded by a mucus layer that prevents direct bacterial contact, which is often compromised in UC, allowing bacterial infiltration and exacerbating inflammation [34]. *Allobaculum*, known to colonize and degrade the mucus layer [35], was significantly enriched in the P and P-UC groups, with a notably higher abundance than in the UC group. This suggests that periodontitis may promote its proliferation, thereby weakening the intestinal barrier and aggravating UC pathology. Although *Allobaculum* can also produce butyrate through glucose fermentation [36, 37], its potential effects on gut health remains unclear and warrants further investigation.

We also observed significant enrichment of *Muribaculum* in the P-UC group, alongside elevated TNF-ɑ and IL-6 levels compared to the UC group. Previous studies have shown that *Muribaculum* metabolites play a key role in upregulating pro-inflammatory cytokines such as TNF-ɑ, IL-6, and IL-23 [38]. These cytokines contribute to inflammation-induced colonic epithelial damage, further disrupting the intestinal barrier. Thus, *Muribaculum* may be another critical player in periodontitis-mediated UC exacerbation. Conversely, *Akkermansia*, a beneficial genus was reduced, potentially diminishing its protective effects. These findings reinforce previous observations and shed light on the intricate, yet underexplored, role of periodontitis in shaping gut microbiota in UC, particularly through its effects on *Allobaculum* and *Muribaculum*.

### Limitations and future directions

Although this study established a clinically relevant chronic UC model and revealed that periodontitis may exacerbate UC by disrupting the intestinal barrier and altering gut microbiota, several limitations should be acknowledged. First, microbial analyses were limited to cecal contents, which may not fully represent microbial diversity across different intestinal regions or timepoints. Longitudinal and spatially resolved sampling in future studies would offer deeper insight into the dynamic progression of gut dysbiosis associated with periodontitis. Second, while previous evidence and our findings suggest a link between periodontitis and UC, the therapeutic implications of periodontal treatment for UC remain unclear. Well-designed randomized controlled trials are needed to determine whether managing periodontitis can improve clinical outcomes in UC patients. Third, the mechanisms underlying the oral-gut axis remain poorly defined. It is unclear whether periodontitis influences UC primarily through the entry of periodontal pathogens or inflammatory mediators into systemic circulation via the ulcerated pocket epithelium [11], or through alterations in the salivary microbiota that subsequently reshape the gut microbial ecosystem [32]. Further studies are needed to clarify whether microbial or immunological pathways dominate this process. Specifically, future research should identify the key bacterial species or consortia involved, or, in the case of immune-driven mechanisms, delineate the critical immune phases, responsible cell subsets, and signaling cascades mediating this interplay.

## Conclusion

Periodontitis may alter the composition of gut microbiota associated with UC, triggering abnormal inflammatory cytokine expression and compromising intestinal barrier integrity—ultimately worsening UC severity. Given this interplay, effective management of periodontitis could play a crucial role in the prevention and treatment of UC. Furthermore, a deeper investigation into the mechanisms by which periodontitis influences UC could offer valuable insights not only into the etiology, diagnosis, and treatment of UC but also into the broader relationship between periodontitis and other systemic diseases.

## Electronic supplementary material

Below is the link to the electronic supplementary material.

Supplementary Material 1

## Data Availability

Sequence data that support the findings of this study have been deposited in the National Library of Medicine Archive with the primary accession code PRJNA1244589 and PRJNA1244561.

## Abbreviations

IBD: Inflammatory bowel disease
UC: Ulcerative colitis
CD: Crohn’s disease
DSS: Dextran sulfate sodium
PCR: Polymerase chain reaction
DAI: Disease activity index
TDI: Tissue damage index
ELISA: Enzyme-linked immunosorbent assay
DAO: Diamine oxidase
CEJ: Cementoenamel junction
ARC: Alveolar crest
TNF-ɑ: Tumor necrosis factor-ɑ
IL-6: Interleukin-6
MCP-1: Monocyte chemoattractant protein-1
ZO-1: Zonula occludens-1
SCFAs: Short-chain fatty acids
