# Supplementary material for: Oral-gut axis in inflammation: periodontitis exacerbates ulcerative colitis via microbial dysbiosis and barrier disruption
Source: BMC Oral Health. 2025 Jun 3;25:894. doi: 10.1186/s12903-025-06269-8 (PMC12135269; doi:10.1186/s12903-025-06269-8)
Supplement: Supplementary file 1 — Supplementary Material 1 [file 12903_2025_6269_MOESM1_ESM.docx]

**Supporting information**

**Oral-Gut Axis in Inflammation: Periodontitis Exacerbates Ulcerative Colitis via Microbial Dysbiosis and Barrier Disruption**

*Jinping Yu* *^#^, Jinglu Lyu^#^, Tongxin Zhu, Yang Li,* *Hanping Xia, Qing Liu,* *Lili Li^*^,* *Bin Chen**^*^*

^#^ Co-first author; ^*^Correspondence

**Affiliation:**

Department of Periodontology, Nanjing Stomatological Hospital, Affiliated Hospital of Medical School, Institute of Stomatology, Nanjing University, Nanjing, China

**Bin Chen:** Department of Periodontology, Nanjing Stomatological Hospital, Affiliated Hospital of Medical School, Institute of Stomatology, Nanjing University, Nanjing, China. E-mail: *[binchen@nju.edu.cn](mailto:binchen@nju.edu.cn); 361734128@qq.com*

**Lili Li:** Department of Periodontology, Nanjing Stomatological Hospital, Affiliated Hospital of Medical School, Institute of Stomatology, Nanjing University, Nanjing, China. E-mail: *njdxlll@163.com*


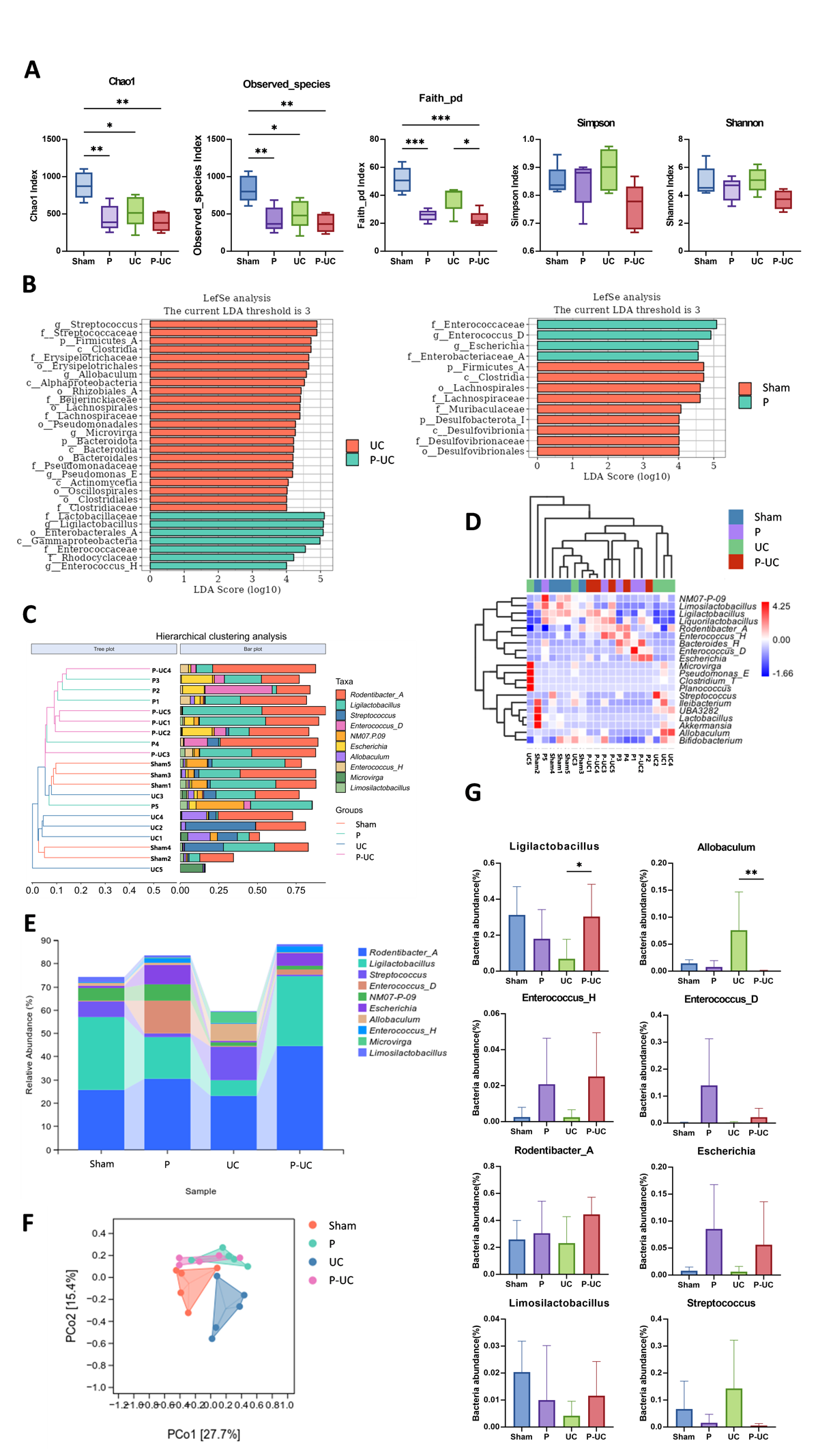


**Figure S1. Effects of periodontitis and colitis on the periodontal microbiota in mice.** **(A)** Alpha diversity indices of periodontal microbiota in each group. Chao1, Observed-species, and Faith-pd indices were significantly lower in the P, UC, and P-UC groups compared to the Sham group. The Faith-pd index was significantly lower in the P-UC group compared to the UC group; **(B)** Differential species and biomarker analysis of periodontal microbiota in each group; **(C)** Hierarchical clustering analysis of periodontal microbiota, showing clear clustering between the UC and P-UC groups; **(D)** UPGMA clustering heatmap of taxonomic composition at the genus level in each group; **(E)** Overview of genus-level composition of periodontal microbiota in each group; **(F)** Distance matrix and PCoA analysis of periodontal microbiota in each group, showing greater distance between the UC and Sham groups and between the UC and P-UC groups, while the P and P-UC groups were closer; **(G)** Quantitative analysis of periodontal microbiota at the genus level. The abundance of *Ligilactobacillus* increased, while *Allobaculum* decreased in the P-UC group compared to the UC group. n=5, **P*< 0.05, ***P*<0.01, ****P*<0.001, *****P*<0.0001. Sham, sham-operated group; P, periodontitis group; UC, UC group; P-UC, periodontitis + UC group.


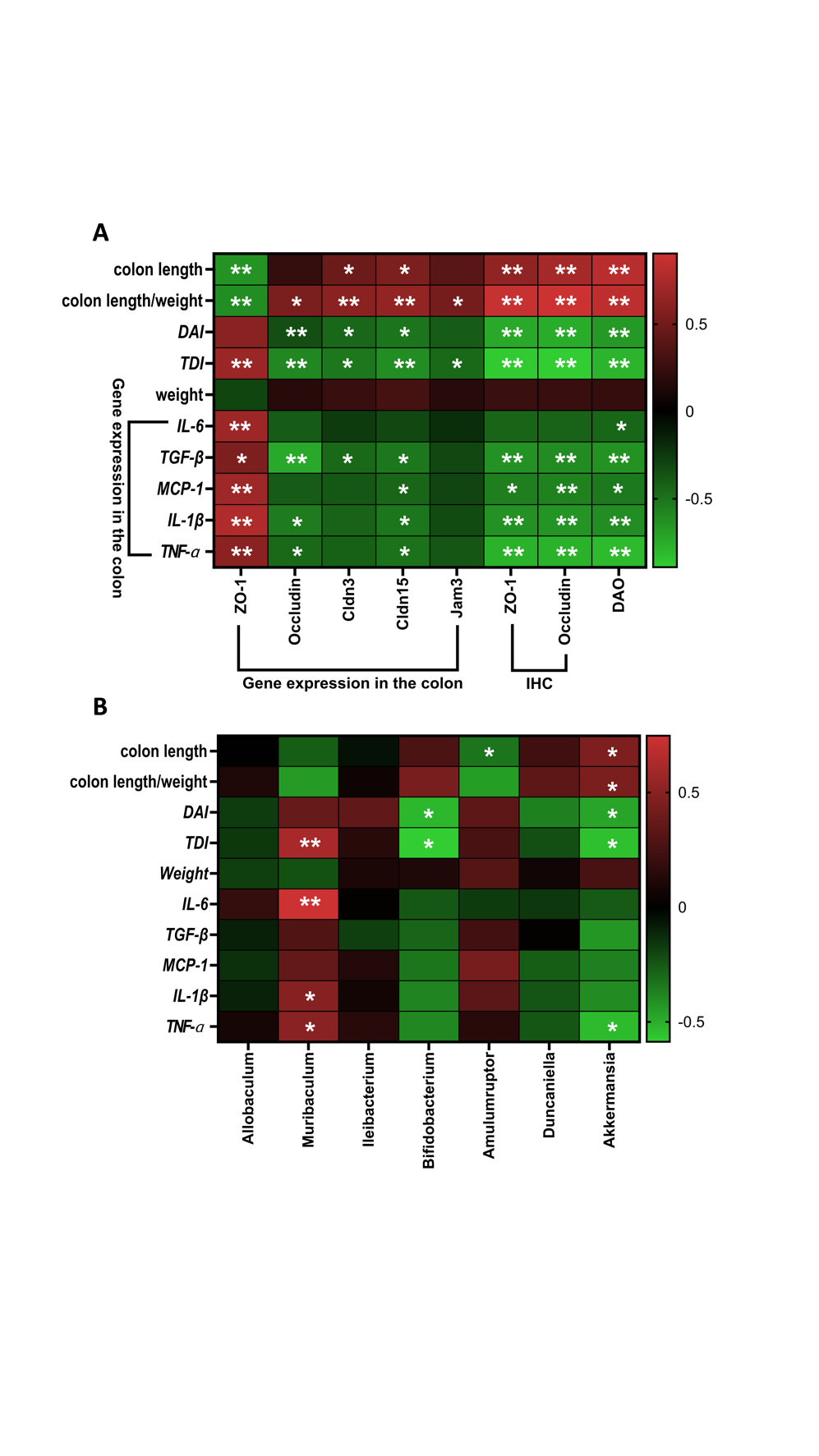


**Figure S2.** **Correlation between** **intestinal barrier,** **gut microbiota and intestinal inflammation.** **(A)** Pearson correlation analysis between intestinal barrier and intestinal inflammation (*P*<0.05, *P*<0.01); **(B)** Pearson correlation analysis between gut microbiota and intestinal inflammation (*P*<0.05, *P*<0.01). **P*< 0.05, ***P* < 0.01.


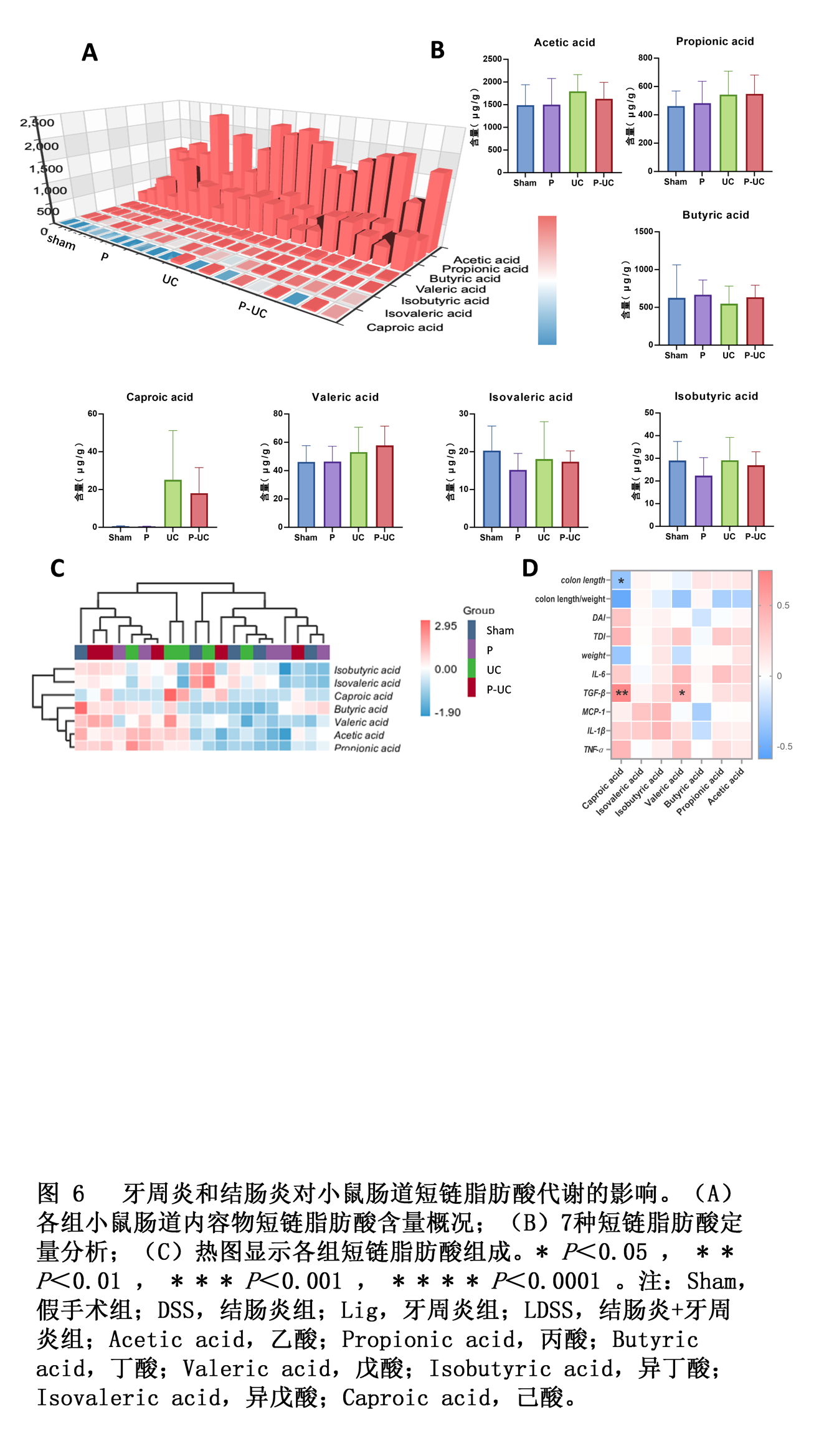


**Figure S3. Effects of periodontitis and colitis on short-chain fatty acids (SCFAs) in mice.** **(A)** Overview of SCFAs content in gut contents, with acetate, butyrate, and propionate accounting for 60%, 20%, and 18%, respectively; **(B)** Quantitative analysis of seven SCFAs. Hexanoic acid levels increased in the UC and P-UC groups compared to the Sham and P groups, but the difference was not significant; **(C)** Heatmap of SCFAs composition in each group; **(D)** Pearson correlation analysis revealed a significant correlation between SCFAs and TGF-β(*P*<0.05). n = 5, **P*< 0.05, ***P* < 0.01, ****P* < 0.001, *****P* < 0.0001. Sham, sham-operated group; P, periodontitis group; UC, UC group; P-UC, periodontitis + UC group.
